# LGBT+ inclusion and human rights in Thailand: a scoping review of the literature

**DOI:** 10.1186/s12889-021-11798-2

**Published:** 2021-10-09

**Authors:** Peter A. Newman, Luke Reid, Suchon Tepjan, Pakorn Akkakanjanasupar

**Affiliations:** 1 Factor-Inwentash Faculty of Social Work, University of Toronto, 246 Bloor Street West, Toronto, Ontario M5S1V4 Canada; 2 VOICES-Thailand Foundation, Chiang Mai, Thailand

**Keywords:** Sexual and gender minorities, LGBT persons, Public nondiscrimination policies, Stigma, Bullying, Marginal employment, Social determinants of health, Health disparities, Scoping review, Thailand

## Abstract

**Background:**

Globally, LGBT+ people continue to struggle to achieve full realization of their human rights. Amid reported health and mental health disparities, and economic insecurity, we conducted a scoping review to explore the breadth of the literature, map and summarize the evidence, and identify knowledge gaps on LGBT+ inclusion and human rights in Thailand.

**Methods:**

We conducted a scoping review in accordance with the methodology developed by the Joanna Briggs Institute and PRISMA-ScR guidelines. We systematically searched 16 databases for peer-reviewed literature, and government and nongovernmental organization websites for grey literature, published in English or Thai from January 1, 2000–August 21, 2020. Two reviewers independently screened studies according to pre-set criteria. We abstracted and analyzed data on publication characteristics and focal populations, and synthesized findings in six domains of LGBT+ inclusion: political and civic participation, education, family, personal security and violence, economic well-being, and health.

**Results:**

The review captured 3327 results in total, which was scoped to 76 peer-reviewed articles and 39 grey literature sources, the majority published after 2010. Gay men and transgender women were the primary focal populations in the peer-reviewed literature, LGBT+ people as a whole in the grey literature. Health was the predominant domain across publications. Key findings include the absence of generalized antidiscrimination legislation for LGBT+ individuals and lack of recourse for transgender individuals to change their legal gender; multifaceted stigma and discrimination in the educational system; social isolation and exclusion in families; disproportionate prevalence of sexual violence and reluctance to report to police; discrimination and marginalization in employment; and LGBT+ disparities in health and mental health.

**Conclusions:**

Future research and programmatic initiatives on LGBT+ inclusion in Thailand should aim to address: 1) understudied populations—lesbian and bisexual women, transmasculine persons; 2) underrepresented topics, including constraints to LGBT+ advocacy; 3) strategic policy initiatives around anti-discrimination laws and legal recognition of same-sex marriage and families; and 4) the need for consistent collection of disaggregated data on LGBT+ persons in education, family, economic, personal security/violence, and health domains in order to assess indicators of inclusion and progress in advancing human rights for LGBT+ people in Thailand.

**Supplementary Information:**

The online version contains supplementary material available at 10.1186/s12889-021-11798-2.

## Background

The inclusion of lesbian, gay, bisexual, and transgender (LGBT), and other persons outside of heteronormative and cisgender identities (i.e., LGBT+) in global society is an essential human rights issue and foundational to health and wellbeing. In 2006, a group of international LGBT+ legal scholars and activists put forward the *Yogyakarta Principles* [1, 2], which recognized persistent “violence, harassment, discrimination, exclusion, stigmatization and prejudice” faced by many LGBT+ persons and articulated a vision for how international human rights principles should be applied in order to counter these invidious social forces and promote full LGBT+ inclusion. Nevertheless, globally, LGBT+ people continue to struggle to achieve full realization of these rights and suffer persistent disparities in terms of physical security [3], economic wellbeing [4], and overall health [5].

The literature on LGBT+ inclusion in Thailand is less extensive than in some western contexts; however, LGBT+ individuals in Thailand are reported to experience poorer health in the form of elevated rates of HIV and AIDS [6], persistent mental health issues like depression and suicidality [7], and greater economic insecurity than heterosexual and cisgender individuals [8]. The general consensus across much of the literature appears to be that many of these disparities are driven by systemic discrimination, social exclusion, and stigma [7, 8], although the limited research on exclusionary and discriminatory forces on LGBT+ populations in Thailand remains fragmented. To this end, we conducted a scoping review to assess the state of the literature as it pertains to LGBT+ inclusion in Thailand.

We approach inclusion as a complex, multi-dimensional concept, grounded in the human development approach pioneered by Amartya Sen [9], and the *Yogyakarta Principles* [1, 2]. Sen focused on “the most basic aspects of self-determination, dignity and freedom” that should be made available to all people ([9], p.8); inclusion is essentially defined as everyone having access to the “capability” to do and be as they choose, and to make choices that lead to outcomes consistent with human dignity. This definition is also embodied in LGBT+ human rights frameworks, such as the *Yogyakarta Principles* [1, 2], which are geared towards achieving freedom and equality through the “full enjoyment of all human rights” (Principle 1). With these frameworks in mind, we utilized the inclusion typology developed by Badgett & Sell [10], which was itself based on Sen’s work, to define the various aspects of inclusion. This typology highlights five key social domains deemed essential to the full inclusion of LGBT+ populations: political and civic participation, education, personal security and violence, economic well-being, and health. For the present review, we added a ‘family’ domain based on our disciplinary training (in social work, psychology, education, public health, and law) and our research and professional experience, which suggest the centrality of family to LGBT+ inclusion.

Notably, a large body of literature addresses “the many forms of gender/sex diversity in Thailand” and the construction of LGBT+ identities (for a review, see Jackson & Duangwises [11], p.1). While the literature on Thai historical and sociocultural contexts of gender and sexuality is related to this review, given the focus of our inquiry, we limited our inclusion criteria to publications that directly addressed concepts of inclusion and human rights, and related “opportunities and outcomes,” as indicated in Badgett & Sell’s ([10], p.1) inclusion framework; this encompassed sociocultural and historical analyses that directly addressed issues around political and civic participation, such as LGBT+ advocacy movements and policy change, or any of the other domains.

The purpose of this scoping review is to explore the breadth of the literature, map and summarize the evidence, and identify knowledge gaps on inclusion and human rights of LGBT+ populations in Thailand. Based on the evidence, we identify directions for future research, program, and policy initiatives.

## Methods

We utilized the scoping review methodology described by Arksey and O’Malley [12], and further developed by the Joanna Briggs Institute [13]. Results are reported in accordance with PRISMA-ScR guidelines [14]. The main steps were the following: 1) identify the purpose of the review and the associated research question; 2) define a search strategy; 3) create a priori inclusion and exclusion criteria; 4) execute the search strategy; 5) chart and synthesize the data; and 6) report the results.

### Research question

This scoping review was guided by the question, “How does the literature describe the inclusion of LGBT+ individuals in Thai society in the following six areas: political and civic participation, education, family, employment, personal security and violence, and health?”

### Information sources and search strategy

We developed the following list of databases to search in consultation with a specialist librarian: Medline, Education Resources Information Centre (ERIC), Applied Social Sciences Index and Abstracts (ASSIA), Public Affairs Information Service Index (PAIS Index), Bibliography of Asian Studies, EconLit, Education Source, Social Work Abstracts, Sociological Abstracts, PsychInfo, LGBTLife, Gender Studies, HeinOnline, ProQuest Thesis, Worldwide Political Science Abstracts, and Child and Adolescent Development. In addition, we conducted targeted searches to locate reports and/or other sources of grey literature including websites of governmental (e.g., Thai Ministry of Social Development and Human Security), quasi-governmental (e.g. United Nations), and local NGO’s (e.g., Rainbow Sky Association of Thailand) (See Additional file 1 for full list).

We used modified versions of a search string previously validated for LGBT+ populations [15] to locate articles. Search strings were then customized to account for the unique syntax of each database surveyed. We also added relevant Thai LGBT+ terminology, including *tom, dee*, *kathoey*, and *sao praphet song*. “*Tom*” (‘butch’), derived from “tomboy” in English, is commonly used to refer to women with masculine gender expression, who are often paired romantically with “*dee*” (‘femme’). *Dee*, a shortened form of “lady” in English, refers to women with feminine gender expression; however, many *toms* and *dees* do not self-identify, nor are they labeled by others, as “lesbian women” [16]. “*Kathoey”* is a commonly used but also contested term for a broad spectrum of transgender persons whose sex is assigned as male at birth but who have a feminine gender identity and/or expression [16, 17]. “*Sao praphet song*”, which literally translates as “women in the second category”, is a term preferred by some transgender women [17]. Given the absence of commonly used Thai language terms for transgender men/transmasculine individuals, which is more recent terminology in the Thai context, only English language terms were used. We also added the term “Thai*” to the search string to limit the results geographically. A sample modified search string is shown in Additional file 2.

We used a truncated search string to identify grey literature as the targeted websites could not accommodate the entire search string above. Following our database search, we reached out to experts (in economics, education, psychology, and law) on LGBT+ inclusion in Thailand or the Asia-Pacific region to review our included publications and to solicit additional sources.

### Study selection criteria

The collection period was for peer-reviewed articles and grey literature sources published between January 1st, 2000 and August 21st, 2020. We developed inclusion and exclusion criteria prior to conducting the search. Publications included in the study had to: 1) focus on LGBT+ individuals or communities; 2) focus on people residing in Thailand (including non-citizens and/or refugees); 3) include research data or analysis on the marginalization, inclusion, discrimination, or comparative well-being of LGBT+ populations.

Publications were excluded from the study if they were 1) published prior to 2000; 2) not written in Thai or English; 3) not focused on LGBT+ populations or people; 4) not focused on individuals residing in Thailand; 5) a review or meta-analysis of other literature; or 6) did not contain primary (qualitative or quantitative) research data or original analysis/commentary.

### Study selection process

Search results of peer-reviewed articles were uploaded into Covidence systematic review software. We held multiple research team discussions to ensure consistent application of inclusion/exclusion criteria. Next, groups of two reviewers (LR, ST, PA) independently screened titles and abstracts for inclusion. All discrepancies were resolved by a third reviewer (PN). Groups of two reviewers (LR and ST/PA) subsequently screened the full text of potentially relevant articles to determine inclusion using the same a priori criteria. All discrepancies between reviewers at the full-text stage were resolved by a single arbitrator (PN). Groups of two reviewers (LR, ST, PA) screened grey literature sources as full texts using the same eligibility criteria and process. Two reviewers agreed on application of the inclusion/exclusion criteria 90% of the time (Cohen’s kappa = 0.69, substantial level of agreement) at the abstract review stage, and 84% of the time (Cohen’s kappa = 0.50, moderate level of agreement) at the full-text screening stage [18].

### Data extraction and synthesis

We abstracted data on publication characteristics (i.e., author(s), year), methods (i.e., qualitative, quantitative, mixed methods, or commentary/descriptive analysis), study sites, focal populations and terminology used, and key study findings about LGBT+ inclusion or human rights (see Table 1). The synthesis included quantitative analysis (e.g., frequency analysis) of the publication year, methods, sites, and focal populations, and qualitative analysis (i.e., content analysis) to determine the domain(s) of LGBT+ inclusion addressed. Two of the authors (LR, ST, PA, PN) identified and categorized the publications by domains of LGBT+ inclusion, with any discrepancies discussed in team meetings and resolved by consensus.

**Table 1:** Study characteristics and domains of inclusion (*n* = 115)

Author(s)	Year	Focal Population(s)	Methods				Domains					
			Quantitative	Qualitative	Mixed	Commentary/ Descriptive	POL	EDUC	VIOL	ECON	HLTH	FAM
**Peer-reviewed Articles (*n*** **= 76)**												
Anand et al. [19]	2017	Young men who have sex with men; Young transgender women		X							X	
Anand et al. [20]	2017	Gay men & other MSM; Transgender women	X								X	
Baral et al. [21]	2014	Gay men & other MSM				X					X	
Beaumont [22]	2006	*Kathoey* (Transgender women)		X			X				X	X
Boer & Emons [23]	2004	General population	X								X	
Burford & Kindon [24]	2015	MSM; Transgender		X			X				X	
Cadoso [25]	2009	Homosexual & other MSM; Bisexual men	X									X
Cadoso [26]	2009	MSM; Bisexual men	X									X
Cadoso & Werner [27]	2013	Heterosexual males	X					X				
Celentano [28]	2005	MSM				X					X	
Chariyalertsak et al. [29]	2011	Gay men; Bisexual men; Transgender women	X								X	
Cheung et al. [30]	2020	General population	X					X				
Claes [31]	2011	*Kathoey* (Transgender women)		X			X			X		X
Closson et al. [32]	2015	Gay men & other MSM; Heterosexuals		X								X
Davis et al. [33]	2019	Transgender women sex workers			X		X	X	X	X	X	X
Dunne et al. [34]	2019	Gay men & other MSM; Transgender women	X							X	X	
Enteen [35]	2007	Lesbian women				X		X				
Fongkaew [36]	2019	LGBT+		X			X		X		X	
Gooren et al. [37]	2013	Transgender women	X								X	X
Gooren et al. [38]	2015	Transgender men; Transgender women	X								X	X
Guadamuz [39]	2007	MSM; MSW; Transgender women	X						X			
Guadamuz et al. [40]	2019	SGM adolescents	X						X		X	
Guadamuz et al. [41]	2015	Young gay men & other MSM		X								X
Guadamuz et al. [42]	2014	Gay men & other MSM	X								X	
Guadamuz et al. [43]	2011	MSM	X						X			X
Hair et al. [44]	2019	Transgender women		X						X		X
Halverson [45]	2017	Transgender women service providers		X					X			
In-iw [46]	2020	Young transgender men & women	X								X	
Jackson [47]	2002	LGBT+				X	X					
Jackson [48]	2011	LGBT+				X	X			X		X
Janyam & Burrows [49]	2013	MSW; Transgender sex workers				X				X	X	
Johnson et al. [50]	2016	Young homosexual & other MSM	X								X	X
Kaeng [51]	2011	LGBT+				X	X					
Käng [52]	2012	*Kathoey* (Transgender women)				X	X			X		X
Käng [53]	2014	LGBT+				X					X	
Khowadhana [54]	2001	Lesbian women		X			X	X		X		X
Khumsaen & Stephenson [55]	2019	MSM			X						X	
Kittiteerasack et al. [56]	2020	LGBT+	X								X	X
Laphon & Chuemchit [57]	2017	Transgender women		X				X		X	X	X
Limaksorn [58]	2018	Lesbian women		X								X
Logie et al. [59]	2016	Young gay men & other MSM; Young transgender women	X						X		X	
Magidson et al. [60]	2016	MSM; Heterosexual men & women	X								X	
Manalastas et al. [61]	2017	General population	X				X	X				
Mutchler [62]	2004	Gay men & other MSM; MSW				X					X	
Nemoto et al. [63]	2016	*Kathoey* (Transgender women)		X						X	X	X
Nemoto et al. [64]	2012	*Kathoey* (Transgender women)			X					X	X	X
Newman et al. [65]	2012	Gay men; Bisexual men; Transgender women	X								X	
Newman et al. [66]	2013	Gay men & other MSM; Transgender women		X							X	
Newman et al. [67]	2012	Gay men & other MSM; Transgender women; General population		X					X		X	X
Noknoi & Wutthirong [68]	2007	LGBT+				X	X		X	X		X
Ocha [69]	2013	Transgender sex workers		X			X			X	X	X
Ojanen [70]	2009	Counsellors to LGBT+		X			X	X	X	X	X	X
Ojanen et al. [71]	2019	LGBT+		X			X	X		X	X	X
Ojanen et al. [72]	2020	LGBT+				X	X	X		X	X	X
Phanuphak et al. [73]	2018	Gay men & other MSM (HIV+)	X								X	
Pongtriaang et al. [74]	2017	Gay men & other MSM; Transgender women		X						X	X	X
Potiwan [75]	2011	Transgender women		X			X			X		
Samakkeekarom [76]	2011	Gay men & other MSM		X			X					
Sanders [77]	2011	LGBT+				X	X		X		X	
Sapsirisavat et al. [78]	2016	Gay men & other MSM	X					X	X	X	X	
Sapsirisavat et al. [79]	2016	Gay men & other MSM	X								X	
Sinnott [80]	2000	LGBT+				X	X	X				
Sinnott [16]	2007	Lesbian women (*Dees, Toms*)				X	X					
Sinnott [81]	2011	LGBT+				X	X				X	
Sopitarchasak et al. [82]	2017	Male adolescents			X			X	X		X	X
Suwatcharapinum [83]	2005	Gay men		X					X		X	
Tangmunkongvorakul et al. [84]	2013	Gay men & other MSM		X							X	X
Thianthai [85]	2019	LGBT+		X				X				
van Griensven et al. [86]	2004	LGBT; General population	X						X		X	X
van Wijngaarden & Fongkaew [87]	2020	Transgender women		X			X	X		X		X
van Wijngaarden & Ojanen [88]	2016	Gay men		X							X	X
Walsh & Chaiyajit [89]	2012	Transgender		X			X					
Winter [90]	2008	*Kathoey* (Transgender women)				X	X			X	X	X
Winter & Udomsak [91]	2002	Transgender women	X							X		
Yadegarfard et al. [92]	2013	Young transgender women	X					X			X	X
Yadegarfard et al. [93]	2014	Young transgender women	X								X	X
**Grey Literature (*n*** **= 39)**												
APCOM [94]	2012	MSM; Transgender people				X					X	
APCOM [95]	2015	MSM				X					X	
APF & UNDP [96]	2016	LGBTI				X	X	X	X	X	X	X
APTN [97]	2017	Trans people				X	X				X	
Beyrer et al. [98]	2011	Gay men & other MSM				X					X	
Cameron [99]	2006	Sex workers; Transgender people; MSM		X			X	X	X	X	X	X
Center for Health Policy Studies, Mahidol University [100]	2016	Students (general population)			X			X	X		X	
Godwin [101]	2010	MSM; Transgender people				X	X		X	X	X	X
ILO [102]	2005	LGBT+				X				X		
Kaleidoscope Human Rights Foundation [103]	2015	LGBTI people				X	X	X	X	X	X	X
Kaleidoscope Human Rights Foundation [104]	2016	LGBTI people				X	X	X			X	
Mahidol University [105]	2014	LGBT students			X			X	X		X	
Newman et al. [106]	2019	LGBTIQ youth			X			X	X		X	X
NHRC [107]	2007	LGBTI people				X	X	X	X	X	X	
OHCHR [108]	2011	LGBT individuals				X	X		X	X	X	X
OHCHR [109]	2018	LGBTIQ people				X	X		X	X		X
Prachatai [110]	2016	LGBTIQ				X		X				
Samakkeekarom & Taesombat [111]	2013	LGBT people			X		X		X			X
Suksom [112]	2020	LGBTI people				X			X			
Suriyasarn [17]	2014	LGBT workers		X			X	X	X	X	X	X
Taengkliang et al. [113]	2015	LGBTI persons				X	X	X	X	X	X	X
Taylor et al. [114]	2017	Migrant workers (general population)		X					X			
The World Bank [115]	2016	General population				X	X	X	X	X	X	X
The World Bank [8]	2018	LGBTI people			X		X	X	X	X	X	X
Tinnam et al. [116]	2019	LGBTIQN+		X			X				X	X
Togetherness for Equality [117]	2017	Women & LBTI persons				X	X	X	X	X	X	X
U.S. Department of State [118]	2011	LGBT				X	X		X	X	X	
U.S. Department of State [119]	2012	LGBT				X	X	X	X	X	X	
U.S. Department of State [120]	2014	LGBT				X	X	X	X	X		
UNDP [7]	2019	LGBT people; Non-LGBT people			X		X	X	X	X	X	X
UNDP & ILO [121]	2018	LGBTI people			X		X			X		
UNDP & Ministry of Social Development and Human Security [122]	2018	LGBTI people		X			X	X			X	
UNDP & USAID [123]	2014	LGBT persons		X			X	X		X	X	X
UNDP [124]	2020	Transgender women		X							X	
UNESCO [125]	2018	LGBTI students		X			X	X	X	X	X	
USAID & UNDP [126]	2011	MSM & Transgender persons				X					X	
van Wijngaarden [127]	2016	Gay men & other MSM				X	X				X	
Vanaspong & Kawesri [128]	2020	Women & LGBT people		X			X					
Winter et al. [129]	2018	Transgender men & women			X		X	X		X		

Note: Terminologies for focal populations are derived from original sources. *ECON* Economic well-being, *EDUC* Education, *FAM* Family, *HLTH* Health, *LGBT+*, Lesbian, gay, bisexual, transgender, *LGBTI* Lesbian, gay, bisexual, transgender, and intersex, *LGBTIQN+* Lesbian, gay, bisexual, transgender, intersex, queer, and non-binary, *MSM* Men who have sex with men, *MSW* Male sex workers, *POL* Political and civic participation, *SGM* Sexual and gender minority, *VIOL* Personal security and violence

## Results

**Fig. 1:**
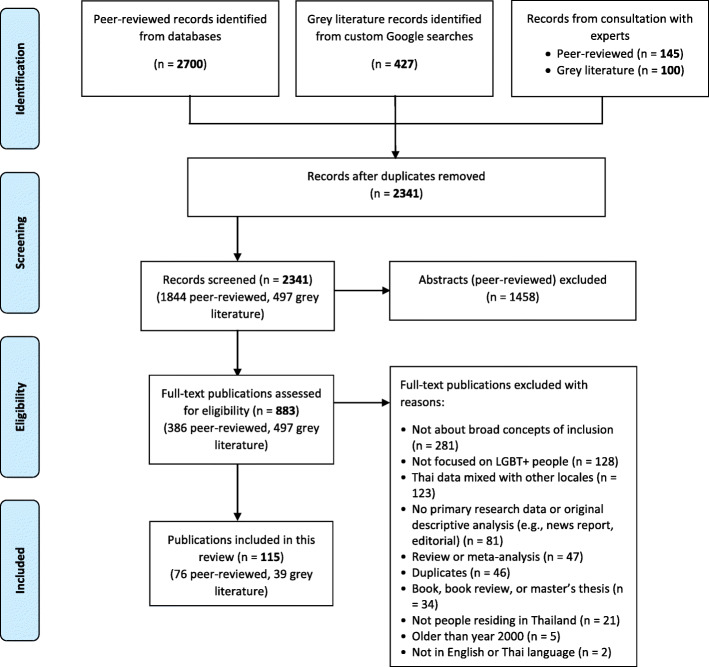
PRISMA flow diagram of study selection process and results

The search captured 1844 peer-reviewed articles and 497 grey literature sources in total. Figure 1 shows the process of identifying relevant peer-reviewed journal articles and grey literature sources. We removed duplicates across the different search engines before reviewing the peer-reviewed article abstracts and the grey literature full texts. After the full text review, we identified a total of 76 [16, 19–93] peer-reviewed articles and 39 [7, 8, 17, 94–129] grey literature sources which were included in the scoping review.

### Description of peer-reviewed studies

Among the 76 peer-reviewed articles included, 37% (28) were quantitative studies [20, 23, 25–27, 29, 30, 34, 37–40, 42, 43, 46, 50, 56, 59–61, 65, 73, 78, 79, 86, 91–93], 36% (27) were qualitative studies [19, 22, 24, 31, 32, 36, 41, 44, 45, 54, 57, 58, 63, 66, 67, 69–71, 74–76, 83–85, 87–89], 22% (17) commentaries or descriptive analyses [16, 21, 28, 35, 47–49, 51–53, 62, 68, 72, 77, 80, 81, 90], and 5% (4) mixed methods studies [33, 55, 64, 82].

Geographically, 81% (48/59) of the quantitative, qualitative, and mixed methods studies were concentrated in urban areas, with Bangkok or Chiang Mai being a primary or sole recruitment site in 88% (42/48) of all urban studies. Only 1 (2%) study took place in rural Thailand, while 5 (8%) were conducted online, and 5 (8%) were regional studies conducted at multiple sites in Thailand.

**Fig. 2:**
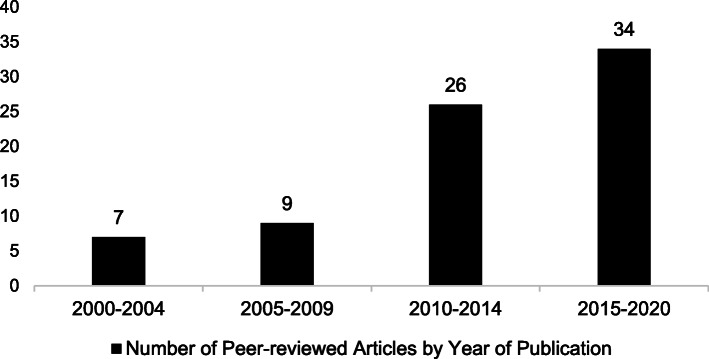
Distribution of peer-reviewed articles by year of publication (*n* = 76)

As Fig. 2 shows, the topic of LGBT+ inclusion in Thailand has been increasing in peer-reviewed articles over the past two decades. The majority (79%; 60/76) of articles were published after 2010.

**Fig. 3:**
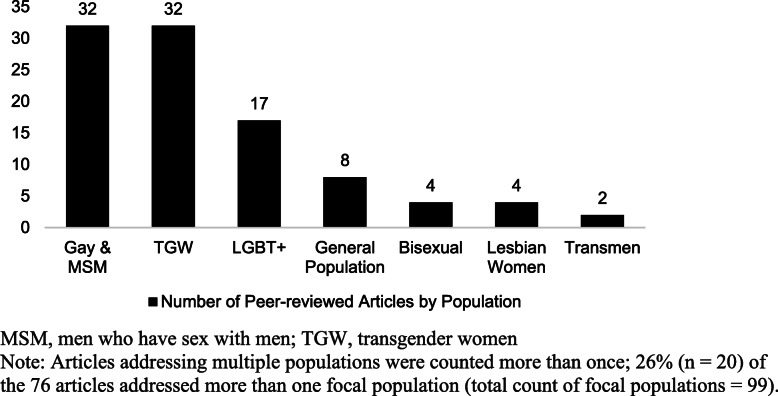
Distribution of focal populations in the peer-reviewed articles

Figure 3 shows the focal populations among the peer-reviewed articles. Across 7 population categories identified, 26% (*n* = 20) of the articles addressed more than one population, including a stated focus on LGBT+ people as a group. Of the 76 articles, 42% (*n* = 32) focussed on gay men or men who have sex with men (MSM); among these, the majority (*n* = 18 articles) used or included the term “gay” or “gay men” (one used “homosexual”), while the remainder only used the behavioral terminology, MSM. As the latter may include men who self-identify as heterosexual, bisexual, or gay, we heretofore refer to the broader category as gay men and other MSM. Thirty-two articles focused on transgender women (12 of these addressed both gay men and transgender populations). Seventeen articles addressed LGBT+ populations broadly, while 4 focussed on bisexual men and women, 4 on lesbian women, and 2 on transmasculine individuals. Eight articles targeted the “general population” or cisgender (i.e., whose gender identity ‘matches’ their sex assigned at birth) heterosexual individuals, largely as a comparator to LGBT+ groups or to gauge their attitudes toward LGBT+ people.

**Fig. 4:**
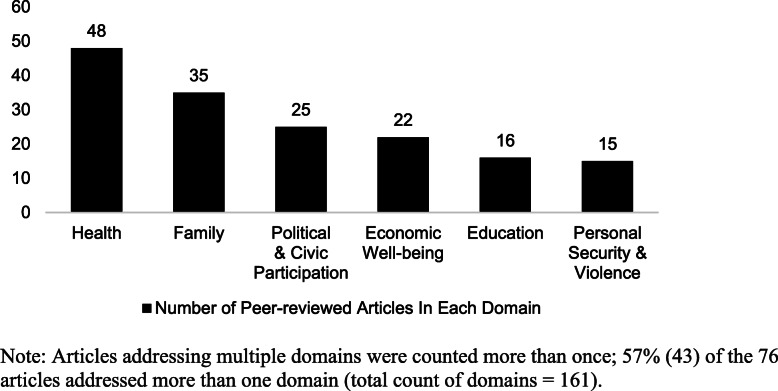
Distribution of peer-reviewed articles by domain of LGBT+ inclusion

Figure 4 shows the number of peer-reviewed articles that addressed each domain of our inclusion typology. Over half (57%; *n* = 43) of the 76 articles addressed multiple domains of inclusion. Accordingly, we identified all domains that were substantively addressed in each article, with a total count of 161. Forty-eight of the articles addressed health, the most prevalent among the six domains of inclusion; 35 articles addressed the family domain and 25 addressed political and civic participation.

### Description of the Grey literature

Of the 39 grey literature sources, 59% (23) were commentaries or descriptive analyses of LGBT+ issues (including NGO reports or legal analyses), 23% (9) utilized qualitative research methods, 3% (1) used quantitative methods, and 15% (6) were large-scale (*n* = 1500+) mixed methods studies primarily conducted by quasi-governmental organizations (e.g., World Bank or UNDP). The focal population of interest of a majority (*n* = 25) of the grey literature sources was LGBT+ people as a group, rather than specific subgroups. Of the remaining grey literature sources, 7 focussed on gay men and other MSM, 5 on transgender persons (2 on transgender women, 1 on transmasculine individuals), 4 targeted the general population, and 1 concentrated on lesbian women, bisexual women, transgender persons and intersex individuals.

The vast majority (97%; 38/39) of the grey literature included was published after 2010; the only exception was a 2007 report by Thailand’s National Human Rights Commission. Geographically, among the 16 empirical studies, 5 were regional and conducted at multiple sites, 4 were conducted in Bangkok, 2 in an unspecified city, 3 online, and 2 were multi-country pan-Asian studies. Over three-quarters of the grey literature sources (77%; *n* = 30) addressed multiple domains of inclusion. Across the total count of 137 domains addressed, 21% (*n* = 29) examined health, similarly the primary domain of focus in the peer-reviewed literature; 20% (*n* = 27) addressed political and civic participation, 17% (*n* = 23) personal security and violence, 15% (*n* = 21) education, 15% (*n* = 21) economic well-being, and 12% (*n* = 16) family.

### Political and civic participation

Overall, the peer-reviewed and grey literature addressing political and civic participation coalesced in the observation that while Thailand “does not conduct active legal repression of sexual/gender minorities”, the law tends to ignore their existence ([70], p.11). This failure to legally acknowledge the existence of LGBT+ individuals creates numerous participatory barriers, two of the most significant examples being the inability to change one’s legal gender and the absence of generalized antidiscrimination legislation for LGBT+ individuals.

Half (27/54) of the publications that addressed political and civic participation, underscored the fact that transgender individuals currently have no recourse to change their legal gender [7, 8, 17, 22, 31, 33, 36, 52, 69–72, 77, 90, 96, 97, 99, 102–104, 113–115, 118, 122–124, 126]. As a result, the gender listed on state-issued identity cards is often incorrect for transgender individuals. This incongruence has been reported to cause numerous issues with respect to employment, foreign travel, and medical services, often acting as a trigger for discriminatory treatment. For example, Suriyasarn [17] reported that as a result of the mismatch between official documentation and physical appearance, hospitals can take significant extra time to verify a transgender individual’s identity, at times causing treatment delays. Similar problems have been reported in accessing government services [8] and social services [124].

The second most-identified challenge (19/52 articles) pertained to the absence of generalized antidiscrimination legislation for LGBT+ individuals [7, 8, 17, 22, 31, 36, 39, 54, 68, 69, 71, 77, 97, 103, 104, 121, 124, 125]. Although the Thai constitution prohibits discrimination on several grounds, including sex, LGBT+ individuals are largely unprotected. The recently enacted *Gender Equality* Act B.E. 2558 [2015] has gone some distance toward remedying this situation for transgender individuals [122, 125]; however, substantial gaps remain as it is “unclear whether this protection extends to sexual orientation” ([8], p.19, 121).

### Education

The 37 publications addressing education as it relates to LGBT+ populations nearly universally underscored the persistent discrimination that LGBT+ individuals face in the Thai education system. A World Bank [8] study (*n* = 2302) indicated that 23% of transgender people, 11% of lesbian women, and 6% of gay men reported that they had experienced discrimination in accessing some form of education or training services. A UNDP [7] study identified even higher prevalence among their sample (*n* = 1349), with 41% of LGBT+ individuals overall reporting that they had experienced discrimination as a student. The proportion for transgender women was the highest, at 61%.

Educational discrimination takes different forms. Fourteen of the 37 sources highlighted bullying and social victimization as significant source of discrimination [7, 8, 33, 40, 71, 82, 87, 96, 103–106, 113, 125]. In a large-scale study [105], the majority (56%) of the LGBT+ individuals (*n* = 2070) experienced bullying in school because of their LGBT+ status. This included verbal/social abuse (e.g., name calling, online bullying, social exclusion), physical abuse (e.g., kicking and slapping), and sexual abuse (e.g., unwanted touching of the breasts, penis or buttocks). A World Bank [8] report conducted in Thailand indicates that this form of abuse is especially concerning because it increases the risk that many LGBT+ students will not finish their schooling, in part, because they may adopt avoidance strategies like skipping school or leaving school altogether. The UNDP [7] noted that over twice as many Thai LGBT+ students who had been bullied (33%) compared to those who had not been bullied (15%) reported unauthorized school absences.

Beyond acts of bullying, many LGBT+ students are subjected to stigmatizing portrayals of LGBT+ populations in school curricula (14/37 articles) [7, 54, 71, 72, 85, 99, 100, 104–106, 110, 113, 123, 125]. In fact, the *2008 Basic Education Core Curriculum* in Thailand covers sexual and gender diversity under the rubric of ‘sexual deviancy’ [100]. This negative portrayal also fuels curricular gaps, including topics related to safer sex among same-sex partners. A UNDP [7] report indicated that nearly two-thirds (64%) of LGBT+ individuals stated that the sex education they had received excluded topics related to sexual orientation and gender identity/expression. Several authors hypothesize that this gap may contribute to the very high rates of HIV infection among young MSM in Thailand [100].

In addition to these curricular gaps, LGBT students reported pressure from teachers to avoid pursuing certain subjects or high-status fields of employment, such as law or medicine, largely based on stereotypical presumptions about their character or ability [8, 129].

Transgender students face unique challenges related to school-wide dress codes (13/37 articles) [7, 70, 71, 87, 100, 103, 104, 113, 120, 125, 126, 128]. Generally, students are required to wear uniforms or have hairstyles that reflect the gender on their identity cards; this can lead to situations where they must either wear clothing that disaffirms their identity or be excluded from their education entirely [70, 71]. Several organizations report that attempts to deviate from the dress code can result in other sanctions, including refusals to allow students to write examinations or submit coursework [103, 129].

### Family

Forty-six (41%) of 115 sources addressed LGBT+ family-related topics, suggesting high relevance to LGBT+ inclusion. The prominence of family may be influenced by the Thai sociocultural context, in contrast to Western countries where family may be less of a fulcrum of life across the lifespan. A majority of these publications (24/46) addressed persistent social isolation, rejection, and discrimination experienced by many LGBT+ individuals in their family despite the powerful significance of family in their lives [7, 17, 25, 32, 33, 37, 38, 44, 56–58, 70, 74, 82, 92, 93, 96, 99, 106, 111, 113, 115, 116, 123]. Several sources specifically underscored the challenges associated with filial obligations and the strong familial pressure to conform that is placed on LGBT+ children in Thai culture [48, 63, 64, 69, 71]. A UNDP [7] national survey (*n* = 1349) indicated that nearly half (48%) of respondents stated that they had experienced at least one form of discrimination within their family. This included pressure to terminate same-sex relationships or enter heterosexual ones, verbal attacks, or being subjected to economic control. A World Bank [115] study (*n* = 868) noted that many LGBT+ individuals reported experiencing violence in their families because of their LGBT+ status. For some LGBT+ youth, familial rejection and discrimination resulted in ejection from the family home [106] and termination of familial support for their education [17]. In a study by Kittiteerasack et al. [56] (*n* = 411), the authors noted high levels of family rejection and reported that approximately half of LGBT+ adults in their study were not out to their family; feeling constrained to hide one’s sexual orientation or gender identity was associated with elevated levels of depression.

Among seven articles that addressed family dynamics related to high rates of rejection, they note substantial pressure is placed on LGBT+ young people to be ‘good’ children and preserve family harmony [54, 69–72, 87, 88]. In some families, being gay is viewed as a defect and there may be intense pressure to adopt heteronormative mannerisms and find an ‘appropriate’ heterosexual partner; failure to conform risks loss of face for the family, and the subsequent rejection of the gay family member [88].

Some LGBT+ individuals appear to manage this pressure with actions rarely described in Western contexts. Five articles indicated that for many LGBT+ individuals, sending money home to their parents, in accordance with filial obligations, can improve familial relationships, in effect “buying” them additional space to express their identity [48, 63, 64, 69, 71]. However, this strategy can have downsides. For example, the limited work and educational opportunities afforded to some transgender individuals means that they are often constrained to engaging in sex work to support their family [63, 64, 71], with its various associated health risks, including HIV infection [63, 64].

Finally, a number of legal hurdles in Thailand prevent LGBT+ individuals from forming their own families. Thirteen publications addressed the difficulties posed by the absence of legal recognition of same-sex marriage [7, 8, 17, 22, 31, 69, 96, 97, 99, 103, 108, 123, 125] and related difficulties in accessing employer pensions and benefits, tax benefits, hospital visitation, and medical decision-making [17, 108]. Gender-specific terms in Sections 1448–1460 of *Part II (Conditions of Marriage)* in *Chapter V (Family)* of the *Civil and Commercial Code* permit marriage only between members of the opposite sex. Many transgender individuals are also barred from marrying, often related to inability to change one’s legal gender [126].

### Personal security and violence

Thirty-five publications addressed threats to the personal security of LGBT+ individuals. This included forced sex and physical violence, and a reluctance to report these instances in the context of police harassment, all of which had negative consequences for LGBT+ people’s health. Twelve studies reported higher rates of forced or coerced sex than among the general population [7, 17, 33, 39, 43, 59, 70, 78, 82, 86, 96, 113], with six-fold higher rates of forced sex reported among young gay and bisexual men and 1.5-fold higher rates among young lesbian and bisexual women (*n* = 1725) compared to their cisgender heterosexual counterparts [86]. A UNDP [7] study (*n* = 1349) reported that 16% of LGBT+ people had been sexually assaulted, with 21% [59], 20% [82], and 18% [43] reported in other studies of gay men and transgender individuals. Ten sources highlighted persistently high rates of physical violence experienced by LGBT+ individuals because of their sexual orientation or gender identity [7, 17, 33, 96, 103, 105, 111, 113, 115, 123]. A World Bank [8] survey of Thailand (*n* = 3502) indicated that 27% of LGBT+ individuals had experienced family violence due to their LGBT+ status, with especially high prevalence (89%) among transgender individuals.

Despite elevated rates of forced sex and violence, LGBT+ people in Thailand are often reluctant to seek assistance from authorities. Thirteen publications identified police harassment and violence as a deterrent to seeking help from authorities [7, 33, 67, 70, 96, 99, 108, 113, 114, 118–120, 126]. Overall, 8% of LGBT+ individuals report harassment by police [7]. Transgender women, especially those engaged in sex work, have been the subject of numerous instances of documented police brutality [33] and are often the target of selective implementation of nuisance and/or vagrancy laws by police [96, 113]. In many cases, police may not take reports of sexual violence against LGBT+ individuals seriously, negating its treatment as criminal conduct [118–120]. Furthermore, Fongkaew et al. [36] and Sinnott [80] described a toxic media environment and suggest that this contributes to a climate in which LGBT+ violence victimization is accepted.

Several articles addressed repercussions of violence in negative health outcomes, including HIV infection [78], illicit drug use [40], depression and suicide [59, 82, 86, 125].

### Economic well-being

Thirty-five publications addressed the economic well-being of LGBT+ people in Thailand, 26 of which highlighted the pervasive discrimination faced by many LGBT+ people in the job market [7, 8, 17, 31, 33, 34, 44, 57, 63, 64, 69–72, 87, 90, 93, 99, 103, 108, 109, 113, 115, 120, 121, 129]. Workplace discrimination takes many different forms, including overly restrictive dress codes that inhibit gender expression [54, 70, 71], mandatory HIV testing as a condition of employment [8, 33, 71], workplace harassment [17, 71, 121], refusing to hire or promote LGBT+ workers because of their gender identity/sexual orientation [17, 121, 129], or simply firing them if their status becomes known [121]. Educational discrimination also exerts a potent impact on job market disparities as it often effectively denies many LGBT+ individuals the necessary qualifications for certain types of work (e.g., law, medicine, etc.) [17, 121]. The *Gender Equality Act* makes it illegal to discriminate “due to the fact that the person is male or female or a different appearance from his/her own sex by birth” (Article 3) ([8], p.9). The fact that Thai law does not appear to explicitly prohibit discrimination on the basis of sexual orientation in the workplace or in the education system [17, 103, 129] may mean that a substantial proportion of LGBT+ individuals are left without recourse in these situations.^1^

A large-scale World Bank [8] study (*n* = 2302) indicated that job discrimination was generally more severe for transgender individuals, 60% of whom reported discrimination in the workplace, compared to 29% of lesbian women and 19% of gay men. Several articles suggest that this disparity is due to the fact that visibly non-normative gender presentation often acts as a trigger for discrimination, with transgender individuals often having more difficulty hiding their LGBT+ status [8, 71, 72]. Indeed, in the World Bank [8] study, the prevalence of hiding one’s identity among different LGBT+ subgroups—41% of gay men, 25% of lesbian women, 23% of transgender individuals—was inversely associated with the prevalence of experiencing workplace discrimination. Nevertheless, the persistent stress of concealing one’s sexual orientation or gender identity and the vigilance it demands, along with anticipated rejection (i.e., minority stress), have negative repercussions for one’s mental health and wellbeing [130]. The fact that Thai law does not currently allow transgender individuals to change their identity documents to reflect their gender further contributes to their being outed as gender non-conforming to potential employers [8, 69, 90, 103, 120, 129].

Overall, the literature on economic well-being suggests that these discriminatory barriers can severely limit employment opportunities for LGBT+ individuals, constraining their opportunities to stereotypical (e.g., hairdressing) or risky professions (e.g., sex work) [75]. Law enforcement, military, religious, and civil service institutions were identified as least accessible, while industries related to beauty and wellness, hospitality, retail, and sex work were among the most accessible [8, 17].

### Health

Health was the most frequently addressed domain, comprising 74 of 115 studies. This was in large measure driven by extensive research literature on HIV: 46 sources addressed HIV risk and related challenges for LGBT+ populations. Moreover, these numbers understate the magnitude of HIV research on LGBT+ populations in Thailand as many HIV-related articles were excluded because they did not address concepts related to inclusion, such as health disparities or discrimination in healthcare.

Many HIV-related publications (13 sources) describe specific risk factors for certain LGBT+ groups, such as *kathoey* sex workers—who experience increased risk as a result of illicit drug use, engaging in condomless anal sex, or having been abused by a father or brother [60]—and MSM, as a result of sex work, drug use, past sexual violence, or experiences of discrimination [34, 78]. These HIV risk factors are exacerbated by systemic discriminatory practices and policies. Several sources implicated stigma in the healthcare system, which creates barriers to HIV testing and prevention [59, 131], as well as a hostile legal environment and police conduct (e.g., raids, harassment etc., during the Social Order Campaign) that can discourage or disrupt safer sex programs [94, 126]. Other scholars highlight the failure of the Thai school system to teach meaningful sex education to LGBT+ students as another driver of high HIV rates among some LGBT+ populations [100, 131].

The relationship between discrimination and poor health outcomes was broadly evident across many sources, including physical health [7, 17, 23, 28, 33, 34, 71, 72, 94, 98, 100, 101, 103, 104, 106, 115, 123, 126, 127] and mental health outcomes. With respect to mental health issues (11 studies), LGBT+ populations are vulnerable to elevated rates of social isolation, depression, and suicidal ideation [34, 40, 56, 82, 86, 92, 93, 116]; negative outcomes are often triggered by discrimination, bullying and/or violence in family, education, economic and/or healthcare domains [7, 8, 105]. A number of sources indicated that one of the primary mediators for this relationship was the poor care and/or discriminatory treatment that many LGBT+ persons receive in the healthcare system. Twenty-six sources called attention to multiple forms of discrimination, spanning indirect to direct, from healthcare providers: inappropriate disclosure of private information or lack of awareness and competence in addressing LGBT+ health issues; applying unequal standards of care to LGBT+ vs. cisgender heterosexuals; characterizing LGBT+ status as a mental illness; to outright refusals to treat LGBT+ people [7, 8, 17–20, 34, 57, 59, 65–67, 69–71, 74, 77–79, 84, 99, 103, 104, 108, 113, 117, 124]. In part, this discrimination may reflect the legacy of the codification of “homosexuality” as a mental illness, delisted by the Thai Ministry of Public Health in 2002—a decision characterized by a former director of the Department of Mental Health as one that “lags behind academic consensus by more than 30 years” [77, 81]. In a study of gay men/MSM and male sex workers (*n* = 260), over one-third (43%) reported that healthcare providers exhibited hostility towards them and 31% reported being given less attention than other patients [65]. A UNDP [7] study indicates that nearly 20% of transgender women reported being refused in-patient accommodation on the women’s hospital ward. Finally, a study of gay men and other MSM, including male sex workers, and transgender women (*n* = 408) found that those who reported higher levels of HIV stigma were 28% less likely to have ever been tested for HIV [59], identifying a direct link between stigma and HIV risk.

Identity cards pose widespread problems in the healthcare context, creating a situation in which transgender people are forced to undergo a demeaning process to prove who they are [17, 103]. The cumulative effect of these barriers is that many LGBT+ individuals avoid the healthcare system because of the widespread, and often accurate, perception that they will be subjected to poor or discriminatory treatment [19, 20, 34, 59, 65–67, 69, 70, 74, 78, 79, 84].

## Discussion

Thailand has a global reputation for LGBT+ tolerance [72]; nevertheless, results of this scoping review demonstrate that LGBT+ individuals face many forms of social exclusion, discrimination, and stigma across multiple domains in Thai society. This includes a legal system that generally ignores LGBT+ populations, an educational system characterized by multifaceted stigma and discrimination against LGBT+ people, and an economic system that constrains LGBT+ people to marginal employment. This paradox between Thailand’s international reputation and the lived experience of many LGBT+ people in Thailand [72] can perhaps be explained by the ambivalence of the Thai public towards LGBT+ rights. Although nearly 70% of the general population report having positive attitudes towards LGBT+ individuals, only 40% indicate support for equal rights and more inclusive policies [7]. This suggests that a large portion of the Thai public opposes the types of measures necessary to remedy the systemic marginalization of LGBT+ individuals.

Despite the public’s limited support for LGBT+ rights, the results of this scoping review indicate broad consensus among scholars and leading intergovernmental organizations and NGOs on the major issues that LGBT+ individuals experience and the policy measures necessary to address these issues. In the domain of political and civic participation, the overwhelming priorities are general LGBT+ antidiscrimination legislation and ability to change one’s legal gender. In education, stigmatizing curricula and persistent bullying are perennial concerns. In the family domain, familial rejection and ostracism in the context of sociocultural beliefs about filial obligation and sexual and gender minority status, and the absence of legal recognition of same-sex marriage are significant concerns.

### Gaps in the literature

Beyond the substantial points of consensus, this scoping review also reveals significant gaps in the existing literature. For one, several NGO’s have criticized what they view as the exclusion of a wide array of LGBT+ health and human rights issues amid a disproportionately high focus and funding for HIV research [103]. Findings from this review corroborate this concern. The subset of peer-reviewed publications focused on HIV *within* the health domain constitute more sources than any of the other five domains of LGBT+ inclusion research.

Overall, several important indicators identified as central to LGBT+ inclusion [10] were largely ignored in scholarship on Thailand or could not be effectively addressed given available resources; these include indicators pertaining to barriers to LGBT+ NGOs operating effectively in a given country. Only three peer-reviewed articles [24, 87, 89] and two grey literature sources [109, 113] addressed challenges associated with LGBT+ advocacy, such as the higher risk of harassment and intimidation that LGBT+ human rights defenders may experience [109, 113]. This critical issue remains largely unstudied.

Other gaps in the literature appear to be related to resources, including available data. Indeed, one consistent recommendation proposed in the broader global literature is the inclusion of questions about LGBT+ groups in official censuses and monitoring mechanisms [7, 8, 17, 113]. Inclusion indicators, such as the relative unemployment rate for LGBT+ people or the relative poverty rate, along with disaggregated data *within* LGBT+ populations, are unlikely to be captured on a consistent and reliable basis in the absence of government support.

Finally, the overrepresentation of gay men and other MSM, and relative underrepresentation of lesbian women, bisexual women and men, intersex individuals, and transmasculine people that emerges in this review is broadly consistent with patterns identified in LGBT+ research literature in other jurisdictions [132–134]. One exception is the relative prominence of transgender women and *kathoeys* in the Thai scholarship, a population generally underrepresented in LGBT+ research in other locales [133, 135–137]. This may speak to the unique cultural status and history of transgender individuals in Thailand [138]. At the same time, the literature addressing marginalization in the economic and education sectors often locates discrimination on the basis of gender expression solely in the context of transgender women; however, discrimination on the basis of visible gender nonconformity also impacts on *tom* and gay men with feminine gender expression [17, 70]. The relative lack of diversity in sexual and gender minority populations included in research in Thailand poses challenges for understanding issues that may be population-specific, and cross-cutting challenges and intersectionalities (e.g., discrimination based on sexual identity *and* gender expression) that impact on LGBT+ inclusion.

Owing in part to the relative abundance of research on HIV and AIDS, and the understandable focus of much of that research on gay, bisexual, and other MSM, and increasingly transgender women, other sexual and gender minority populations at lower risk for HIV infection remain understudied. Within the health domain, other potential disparities among LGBT+ populations—including lesbian and bisexual women, and transgender men—merit increased attention, such as in the areas of tobacco, alcohol, and other substance use, and mental health in the context of multilevel stigma and discrimination [72]. Further research is also warranted with diverse sexual and gender minority populations beyond the health domain, to understand their unique perspectives, challenges, and successes in gaining full acceptance and inclusion in Thai society. To that end, collection and disaggregation of data by gender and sexual identity, such as in future implementation of the Thai Demographic and Health Survey (TDHS), is crucial to advancing LGBT+ inclusion.

### Indivisibility and interrelatedness

A number of important implications for LGBT+ human rights and inclusion as a whole can be drawn from this scoping review. The broader literature amply demonstrates an empirical foundation for the principle that all human rights are fundamentally interrelated and indivisible [139]. This principle is embedded in the Yogyakarta Principles [1] and the conceptual definition of inclusion itself, which focuses on maximizing people’s capacities to be and do as they choose in multiple areas of life [9]. The fact that almost half of the articles engaged with at least two or more domains of inclusion illustrates the interconnectedness of these issues and the difficulty of contending with any one domain in isolation. Significantly, it also illustrates how failure to respect the human rights of LGBT+ individuals in one domain reverberates through many others, ultimately impairing the ability to define the parameters of one’s life and live to one’s full potential.

The issue of identity cards for transgender individuals provides an apt example of the interconnectedness among the domains of inclusion. Failure of the Thai legal system to allow transgender persons to change their legal gender perpetuates discrimination in the Thai education system through the application of restrictive dress codes; in the labour market, by ‘outing’ transgender persons to their (prospective) employers; and in the healthcare system when it comes to having to prove their identity in order to access care. This acts as a trigger for discrimination throughout Thai society, limiting educational and economic opportunities, and constraining many transgender persons to accepting only risky informal work, such as sex work, with its elevated risk of contracting HIV and of violence. Ultimately, the pattern that emerges is that one rights violation often precipitates another—a domino effect that not only operates across domains but can exert an impact across one’s lifetime: early experiences of marginalization and exclusion, if not remedied or addressed, can impact LGBT+ youth from early adolescence well into old age.

The UNDP [7] aptly captures the indivisibility of these domains of inclusion in discussing mental health challenges faced by LGBT+ youth in Thailand, described as “a function of stigma and rejection…that occur across school, family, and healthcare settings. It is not necessarily any one event, or one sector, that emerges as primary; rather it is the pervasiveness of stigma across multiple key domains of [a] youth’s social ecology….” This is evident in several studies in this review in the health domain, which indicate that experiences of discrimination by LGBT+ people are significantly statistically associated with barriers to healthcare access and negative health outcomes [34, 59, 64, 78].

### Considerations for expanding the inclusion framework

This scoping review also suggests that it may be useful to expand the inclusion typology developed by Badgett & Sell [10] to include a ‘family’ domain. The sheer number of publications (46/115) that address family suggests its relevance to the concept of inclusion, and moreover that it may be a useful indicator in Thailand. The family sector emerged as important in its own right and as a determinant for other types of inclusion. Family inclusion was found to have a substantial impact on the educational prospects of LGBT+ individuals, their overall health, and importantly represented a powerful source of acceptance for many LGBT+ people. The family domain also encompasses several significant policy and advocacy issues for LGBT+ inclusion; its delineation may serve as a heuristic, encouraging further investigation of familial inclusion, as well as serving a symbolic function in promoting inclusive conceptualizations of “family” rather than ceding it as a heteronormative construction.

Although not reported in our results, 15/115 publications addressed religious aspects of inclusion; in the Thai context, this emphasizes how LGBT+ status is interpreted through the lens of Theravada Buddhism [22, 31, 33, 34, 52, 61, 63, 64, 69, 70, 72, 87, 93, 99]. This suggests that a religious domain for inclusion may be of interest to scholars and policy advocates, and deserving of further investigation. An understanding of the role of religious sociocultural contexts in LGBT+ inclusion may support not only an understanding of potential barriers to inclusion, but of strategic opportunities to advance inclusion and human rights.

### Limitations

The present findings should be understood in the context of the limitations of this review. First, given the multisectoral nature of this review, an expansive number of sources may have some bearing on the findings even as they do not specifically address LGBT+ discrimination, social exclusion, or disparities; some of these may have been omitted in accordance with our a priori specification of inclusion criteria. However, we conducted an extensive and rigorous review of 115 peer-reviewed articles and grey literature sources that yielded many consistent and well-supported findings. Second, while the inclusion of grey literature increased the comprehensiveness of the review, it also leaves open the possibility that biased or substandard material may have been included. However, we thoroughly screened the grey literature for inclusion, and it yielded several large-scale mixed methods studies. Overall, the findings from the grey literature corroborate and expand on those from the peer-reviewed literature. Third, by design we included both Thai- and English-language literature; nevertheless, database functionalities and lack of inclusion of Thai domestic journals in internationally recognized databases delimit the Thai literature available. Our involvement of bilingual Thai-English co-authors and experts consulted supported inclusion and expanded the breadth of Thai language sources.

Fourth, while the collaboration of reviewers and coauthors who are Thai and English native speakers was an asset, it may have contributed to the moderate level of reviewer agreement at the full-text stage. Nevertheless, reviewers were trained in application of inclusion/exclusion criteria and all discrepancies were resolved by a single arbitrator, the primary author. We report both percent reviewer agreement and Cohen’s kappa, as recommended, considering what may be overly conservative assumptions underlying the kappa statistic [140]. Finally, sexual and gender minority identities and categories are dynamic, contested, and influenced by culture, history, and politics [16, 70, 138]. To the extent possible, we reflected the identities and labels used in original sources and made explicit our use of terminology with the goal of synthesizing the literature into meaningful observations and actionable recommendations to support LGBT+ inclusion.

## Conclusions

The literature on LGBT+ inclusion in Thailand is growing rapidly. The majority of peer-reviewed articles (79%; 60/76) and grey literature sources (97%; 38/39) in this scoping review were published after 2010. In this review, we provide a comprehensive examination and synthesis of the scholarly consensus on LGBT+ inclusion in Thailand, and we identify important gaps in the literature. Our review also demonstrates a compelling case for the utility of Badgett and Sell’s [10] LGBT+ inclusion framework, and for expanding it to encompass a sixth domain, namely family. As the results of this review indicate, future research on LGBT+ inclusion in Thailand should aim to address: 1) current gaps in the literature, especially those pertaining to understudied populations, such as lesbian and bisexual women, and transmasculine persons; 2) underrepresented topics, such as constraints to LGBT+ advocacy; 3) strategic policy initiatives, such as anti-discrimination laws and legal recognition of same-sex marriage and families; and 4) the need for consistent collection of disaggregated data on sexual and gender identity pertinent to each domain in order to assess indicators of inclusion and progress in advancing human rights for LGBT+ people in Thailand.

## Supplementary Information

**Additional file 1.**

**Additional file 2.**

## Data Availability

All data generated or analysed during this study are included in this published article.

## Abbreviations

HIV: Human immunodeficiency virus
LGBT: Lesbian, gay, bisexual, and transgender persons
LGBT+: LGBT and other persons outside of heteronormative and cisgender identities (e.g., queer, intersex, asexual, nonbinary, genderfluid, pansexual, polyamorous, questioning)
MSM: Men who have sex with men
NGO: Non-governmental organisation
UNDP: United Nations Development Programme

## Footnotes

1 Article 5.6 of the *Thai Labour Standard* (TLS 8001–2010) does purport to prohibit discrimination with respect to employment, promotion, termination, payment of wages/benefits, and training opportunities based on the “personal attitude on gender or sexual orientation”; however, this is a voluntary standard and does not carry the force of law. Other statutes, like the *Civil Service Act*, actually appear to create significant opportunities for discrimination during the hiring process. Section 36 provides that individuals who are “morally defective to the extent of being socially objectionable” are prohibited from employment within the civil service. (See: Suriyasarn, 2015 [17] & NATLEX – ILO Database of International Labour, Social Security and Human Rights Legislation; https://www.ilo.org/dyn/natlex/natlex4.home)

## References

[1] Yogyakarta Principles (2006). Principles on the application of human rights law in relation to sexual orientation and gender identity.

[2] Yogyakarta Principles plus 10 (2017). Principles on the Application of Human Rights Law in relation to Sexual Orientation and Gender Identity.

[3] Mendos LR (2019). State-sponsored homophobia.

[4] Badgett MV, Nezhad S, Waaldijk K, van der Meulen Rodgers Y (2014). The relationship between LGBT inclusion and economic development: an analysis of emerging economies 2014.

[5] Rodríguez-Díaz CE, Martínez-Vélez JJ, Jovet-Toledo GG, Vélez-Vega CM, Hernández-Otero N, Escotto-Morales B, Mulinelli-Rodríguez JJ (2016). Challenges for the well-being of and health equity for lesbian, gay, and bisexual people in Puerto Rico. Int J Sexual Health.

[6] UNAIDS (2018). Country Report – Thailand.

[7] UNDP (2019). Tolerance but not Inclusion: a national survey on experiences of discrimination and social attitudes towards LGBT people in Thailand.

[8] The World Bank (2018). Economic inclusion of LGBTI groups in Thailand.

[9] UNDP (2016). Measuring LGBTI inclusion: increasing access to data and building the evidence base.

[10] Badgett ML, Sell R (2018). A set of proposed indicators for the LGBTI inclusion index.

[11] Jackson PA, Duangwises N (2017). Review of studies of gender and sexual diversity in Thailand in Thai and international academic publications [Preceding]. 13th International Conference on Thai Studies, July 2017.

[12] Arksey H, O’Malley L (2005). Scoping studies: towards a methodological framework. Int J Soc Res Methodol.

[13] Joanna Briggs Institute (2015). Joanna Briggs institute reviewers’ manual: 2015 edition/ supplement.

[14] Tricco AC, Lillie E, Zarin W, O'Brien KK, Colquhoun H, Levac D, Moher D, Peters MDJ, Horsley T, Weeks L, Hempel S, Akl EA, Chang C, McGowan J, Stewart L, Hartling L, Aldcroft A, Wilson MG, Garritty C, Lewin S, Godfrey CM, Macdonald MT, Langlois EV, Soares-Weiser K, Moriarty J, Clifford T, Tunçalp Ö, Straus SE (2018). PRISMA extension for scoping reviews (PRISMA-ScR): checklist and explanation. Ann Intern Med.

[15] Lee JG, Ylioja T, Lackey M (2016). Identifying lesbian, gay, bisexual, and transgender search terminology: a systematic review of health systematic reviews. PLoS One.

[16] Sinnott M, Wieringa SE, Blackwood E, Bhaiya A (2007). Gender subjectivity: dees and toms in Thailand. Women’s sexualities and masculinities in a globalizing Asia–comparative feminist studies series.

[17] Suriyasarn B (2014). Gender identity and sexual orientation in Thailand: promoting rights, diversity, and equality in the world of work (PRIDE) project.

[18] Landis JR, Koch GG (1977). The measurement of observer agreement for categorical date. Biometrics.

[19] Anand T, Nitpolprasert C, Kerr SJ, Muessig KE, Promthong S, Chomchey N, Hightow-Weidman LB, Chaiyahong P, Phanuphak P, Ananworanich J, Phanuphak N (2017). A qualitative study of Thai HIV-positive young men who have sex with men and transgender women demonstrates the need for eHealth interventions to optimize the HIV care continuum. AIDS Care.

[20] Anand T, Nitpolprasert C, Trachunthong D, Kerr SJ, Janyam S, Linjongrat D, Hightow-Weidman LB, Phanuphak P, Ananworanich J, Phanuphak N, Adam's Love study team (2017). A novel online-to-offline (O2O) model for pre-exposure prophylaxis and HIV testing scale up. J Int AIDS Soc.

[21] Baral SD, Grosso A, Holland C, Papworth E (2014). The epidemiology of HIV among men who have sex with men in countries with generalized HIV epidemics. Curr Opin HIV AIDS.

[22] Beaumont A (2006). ‘Betwixt and Between’: a comparative study of the transgender experience in Britain and Thailand [dissertation].

[23] Boer H, Emons PAA (2004). Accurate and inaccurate HIV transmission beliefs, stigmatizing and HIV protection motivation in northern Thailand. AIDS Care.

[24] Burford J, Kindon S (2015). Queering accounts of “MSM” practitioner agency: recognising collateral benefits. Dev Pract.

[25] Cardoso FL (2009). Recalled sex-typed behavior in childhood and sports’ preferences in adulthood of heterosexual, bisexual, and homosexual men from Brazil, Turkey, and Thailand. Arch Sex Behav.

[26] Cardoso FL (2010). Political and sexual attitudes concerning same-sex sexual behavior. Sex Cult.

[27] Cardoso FL, Werner D (2013). Same-sex behavior of heterosexual men: a cross-cultural comparison. J Bisex.

[28] Celentano DD (2005). Why has the Thai HIV epidemic in men who have sex with men been so silent?. AIDS.

[29] Chariyalertsak S, Kosachunhanan N, Saokhieo P, Songsupa R, Wongthanee A, Chariyalertsak C, Visarutratana S, Beyrer C (2011). HIV incidence, risk factors, and motivation for biomedical intervention among gay, bisexual men, and transgender persons in northern Thailand. PLoS One.

[30] Cheung DH, Boonmongkon P, Ojanen TT, Damri T, Samoh N, Cholratana M, Ratchadapunnathikul C, Gilman SE, Sass J, Guadamuz TE (2020). Peer victimisation and depression among gender conforming and non-conforming Thai adolescents. Cult Health Sex.

[31] Claes MT (2011). Kathoeys of Thailand: a diversity case in international business. Int J Divers Organ Communities Nations.

[32] Closson EF, Mimiaga MJ, Sherman SG, Tangmunkongvorakul A, Friedman RK, Limbada M, Moore AT, Srithanaviboonchai K, Alves CA, Roberts S, Oldenburg CE, Elharrar V, Mayer KH, Safren SA, for the HPTN063 study team (2015). Intimacy versus isolation: a qualitative study of sexual practices among sexually active HIV-infected patients in HIV care in Brazil, Thailand, and Zambia. PLoS One.

[33] Davis J, Quinley J, Miles G (2019). “Same same, but different”: a baseline study on the vulnerabilities of transgender sex workers in Bangkok’s sex industry. Int J Sociol Soc Policy.

[34] Dunne EF, Pattanasin S, Chemnasiri T, Varangrat A, Raengsakulrach B, Wichuda S, Ungsedhapand C, Sirivongrangson P, Chitwarakorn A, Holtz TH (2019). Selling and buying sex in the city: men who have sex with men in the Bangkok men who have sex with men cohort study. Int J STD AIDS.

[35] Enteen J (2007). Lesbian studies in Thailand. J Lesbian Stud.

[36] Fongkaew K, Khruataeng A, Unsathit S, Khamphiirathasana M, Jongwisan N, Arlunaek O, Byrne J (2019). “Gay guys are shit-lovers” and “lesbians are obsessed with fingers”: the (mis)representation of LGBTIQ people in Thai news media. J Homosex.

[37] Gooren LJ, Sungkaew T, Giltay EJ (2013). Exploration of functional health, mental well-being and cross-sex hormone use in a sample of Thai male-to-female transgendered persons (kathoeys). Asian J Androl.

[38] Gooren LJ, Tanapong S, Giltay EJ, Guadamuz TE (2015). Cross-sex hormone use, functional health and mental well-being among transgender men (toms) and transgender women (kathoeys) in Thailand. Cult Health Sex..

[39] Guadamuz TE (2007). Using venue-day-time sampling to assess HIV prevalence and correlates among men who have sex with men populations in Thailand [dissertation].

[40] Guadamuz TE, Cheung DH, Boonmongkon P, Ojanen TT, Damri T, Samoh N, Cholratana M, Ratchadapunnathikul C, Sass J (2019). Illicit drug use and social victimization among Thai sexual and gender minority adolescents. Subst Use Misuse.

[41] Guadamuz TE, Goldsamt LA, Boonmongkon P (2015). Consent challenges for participation of young men who have sex with men in HIV prevention research in Thailand. Ethics Behav.

[42] Guadamuz TE, McCarthy K, Wimonsate W, Thienkrua W, Varangrat A, Chaikummao S, Sangiamkittikul A, Stall RD, van Griensven F (2014). Psychosocial health conditions and HIV prevalence and incidence in a cohort of men who have sex with men in Bangkok, Thailand: evidence of a syndemic effect. AIDS Behav.

[43] Guadamuz TE, Wimonsate W, Vanrangrat A, Phanuphak P, Jommaroen R, Mock PA (2011). Correlates of forced sex among populations of men who have sex with men in Thailand. Arch Sex Behav.

[44] Hair SA, King J, Edwards N, Hayes S (2019). Older transgender women in Thailand: views of service providers. J Gay Lesbian Soc Serv.

[45] Halverson AM (2017). Intimate partner victimization of transgender people and access to social services [dissertation].

[46] In-iw S (2020). Non-prescribed cross-sex hormone use and risky behaviors among Thai transgender youth. J Adolesc Health.

[47] Jackson PA, Heng RHK (2002). Offending images: gender and sexual minorities, and state control of the media in Thailand. Media fortunes, changing times: ASEAN states in transition.

[48] Jackson PA, Jackson PA (2011). Capitalism, LGBT activism, and queer autonomy in Thailand. Queer Bangkok: twenty-first-century markets, media and rights.

[49] Janyam KS, Burrows N (2013). Male and trans* sex workers self-organise against stigma, discrimination and HIV in Thailand. HIV Australia.

[50] Johnston LG, Steinhaus MC, Sass J, Sirinirund P, Lee C, Benjarattanaporn P, Gass R (2016). Recent HIV testing among young men who have sex with men in Bangkok and Chiang Mai: HIV testing and prevention strategies must be enhanced in Thailand. AIDS Behav.

[51] Kaeng DB (2011). Queer media loci in Bangkok: paradise lost and found in translation. GLQ.

[52] Käng DB (2012). Kathoey “In trend”: emergent genderscapes, national anxieties and the re-signification of male-bodied effeminacy in Thailand. Asian Stud Rev.

[53] Käng DB, Liamputtong P (2014). Conceptualizing Thai genderscapes: transformation and continuity in the Thai sex/gender system. Contemporary socio-cultural and political perspectives in Thailand.

[54] Khowadhana S (2001). Hermeneutics of understanding women in Thailand [dissertation].

[55] Khumsaen N, Stephenson R (2019). Feasibility and acceptability of an HIV/AIDS self-management education program for HIV-positive men who have sex with men in Thailand. AIDS Educ Prev.

[56] Kittiteerasack P, Steffen A, Matthews A (2020). The influence of minority stress on level of depression among Thai LGBT adults. Jurnal Keperawatan Indonesia.

[57] Laphon C, Chuemchit M (2017). Social stigmatization access to services and service satisfaction among transgender persons at Thai red cross AIDS research center - tangerine project: a qualitative study. J Health Res.

[58] Limaksorn K (2018). From “purple” to “rainbow”: re-defining lesbianism in Thailand [dissertation].

[59] Logie CH, Newman PA, Weaver J, Roungkraphon S, Tepjan S (2016). HIV-related stigma and HIV prevention uptake among young men who have sex with men and transgender women in Thailand. AIDS Patient Care STDs.

[60] Magidson JF, Li X, Mimiaga MJ, Moore AT, Srithanaviboonchai K, Friedman RK, Limbada M, Hughes JP, Cummings V, Gaydos CA, Elharrar V, Celentano D, Mayer KH, Safren SA (2016). Antiretroviral medication adherence and amplified HIV transmission risk among sexually active HIV-infected individuals in three diverse international settings. AIDS Behav.

[61] Manalastas EJ, Ojanen TT, Torre BA, Ratanashevorn R, Hong BCC, Kumaresan V (2017). Homonegativity in Southeast Asia: attitudes toward lesbians and gay men in Indonesia, Malaysia, the Philippines, Singapore, Thailand and Vietnam. Asia-Pacific Soc Sci Rev.

[62] Mutchler MG (2004). Money-boys in Thailand: sex, work, and stigma during the XV international AIDS conference. J HIV AIDS Prev Child Youth.

[63] Nemoto T, Cruz T, Iwamoto M, Trocki K, Perngparn U, Areesantichai C, Suzuki S, Roberts C (2016). Examining the sociocultural context of HIV-related risk behaviors among kathoey (male-to-female transgender women) sex workers in Bangkok, Thailand. J Assoc Nurses AIDS Care.

[64] Nemoto T, Iwamoto M, Perngparn U, Areesantichai C, Kamitani E, Sakata M (2012). HIV-related risk behaviors among kathoey (male-to-female transgender) sex workers in Bangkok. Thailand AIDS Care.

[65] Newman PA, Lee SJ, Roungprakhon S, Tepjan S (2012). Demographic and behavioral correlates of HIV risk among men and transgender women recruited from gay entertainment venues and community-based organizations in Thailand: implications for HIV prevention. Prev Sci.

[66] Newman PA, Roungprakhon S, Tepjan S (2013). A social ecology of rectal microbicide acceptability among young men who have sex with men and transgender women in Thailand. J Int AIDS Soc.

[67] Newman PA, Roungprakhon S, Tepjan S, Yim S, Walisser R (2012). A social vaccine? Social and structural contexts of HIV vaccine acceptability among most-at-risk populations in Thailand. Glob Public Health.

[68] Noknoi C, Wutthirong P (2007). Workforce diversity: sexual orientation discrimination in Thailand. Int J Divers Organ Communities Nations.

[69] Ocha W (2013). Rethinking gender: negotiating future queer rights in Thailand. Gend Technol Dev.

[70] Ojanen TT (2009). Sexual/gender minorities in Thailand: identities, challenges, and voluntary-sector counseling. Sex Res Soc Policy.

[71] Ojanen TT, Burford J, Juntrasook A, Kongsup A, Assatarakul T, Chaiyajit N (2019). Intersections of LGBTI exclusion and discrimination in Thailand: the role of socio-economic status. Sex Res Soc Policy.

[72] Ojanen TT, Newman PA, Ratanashevorn R, van Wijngaarden JWDL, Tepjan S, Nakamura N, Logie CH (2020). Whose paradise? An international perspective on mental health and gender/sexual diversity in Thailand. LGBT mental health: international perspectives and experiences.

[73] Phanuphak N, Anand T, Jantarapakde J, Nitpolprasert C, Himmad K, Sungsing T, Trachunthong D, Phomthong S, Phoseeta P, Tongmuang S, Mingkwanrungruang P, Meekrua D, Sukthongsa S, Hongwiangchan S, Upanun N, Barisri J, Pankam T, Phanuphak P (2018). What would you choose: online or offline or mixed services? Feasibility of online HIV counselling and testing among Thai men who have sex with men and transgender women and factors associated with service uptake. J Int AIDS Soc.

[74] Pongtriang P, O’Brien AP, Maguire J (2017). Shame and blame and its influence on male gay (chaay rak chaay) quality of life in Bangkok Thailand: a health promotion community nursing perspective. J Public Ment Health.

[75] Potiwan P (2011). Identity, sub-cultural and social space of the transgender. Damrong J.

[76] Samakkeekarom R, Jackson PA (2011). Cyberspace, power structures, and gay sexual health: the sexuality of Thai men who have sex with men (MSM) in the Camfrog on-line web cam chat rooms. Queer Bangkok: twenty-first-century markets, media and rights.

[77] Sanders D, Jackson PA (2011). The rainbow lobby: the sexual diversity network and the military-installed government in Thailand. Queer Bangkok: 21st century markets, media, and rights.

[78] Sapsirisavat V, Phanuphak N, Keadpudsa S, Egan JE, Pussadee K, Klaytong P (2016). Psychosocial and behavioral characteristics of high-risk men who have sex with men (MSM) of unknown HIV positive serostatus in Bangkok, Thailand. AIDS Behav.

[79] Sapsirisavat V, Phanuphak N, Sophonphan J, Egan JE, Langevattana K, Avihingsanon A (2016). Differences between men who have sex with men (MSM) with low CD4 cell counts at their first HIV test and MSM with higher CD4 counts in Bangkok, Thailand. AIDS Behav.

[80] Sinnott M (2000). The semiotics of transgendered sexual identity in the Thai print media: imagery and discourse of the sexual other. Cult Health Sex.

[81] Sinnott M, Jackson PA (2011). The language of rights, deviance, and pleasure: organizational responses to discourses of same-sex sexuality and transgenderism. Queer Bangkok: twenty-first century markets, media, and rights.

[82] Sopitarchasak S, Kihara M, Soe KM, Ono-Kihara M (2017). Disparities in mental well-being between non-minority and sexual minority male youth in Bangkok, Thailand: quantitative findings from a mixed method study. J Popul Soc Stud.

[83] Suwatcharapinum S (2005). Spaces of male prostitution: tactics, performativity and gay identities in streets, go-go bars and magazines in contemporary Bangkok, Thailand [dissertation].

[84] Tangmunkongvorakul A, Chariyalertsak S, Amico KR, Saokhieo P, Wannalak V, Sangangamsakun T, Goicochea P, Grant R (2013). Facilitators and barriers to medication adherence in an HIV prevention study among men who have sex with men in the iPrEx study in Chiang Mai, Thailand. AIDS Care.

[85] Thianthai C (2019). Young people's views of the constraints on sex education in Bangkok, Thailand. Sex Educ.

[86] van Griensven F, Kilmarx PH, Jeeyapant S, Manopaiboon C, Korattana S, Jenkins RA, Uthaivoravit W, Limpakarnjanarat K, Mastro TD (2004). The prevalence of bisexual and homosexual orientation and related health risks among adolescents in northern Thailand. Arch Sex Behav.

[87] van Wijngaarden JWDL, Fongkaew K. “Being born like this, I have no right to make anybody listen to me”: understanding different forms of stigma among Thai transgender women living with HIV in Thailand. J Homosex. 2020. [epub ahead of print]:1–18. 10.1080/00918369.2020.1809892. 10.1080/00918369.2020.1809892 32841090

[88] van Wijngaarden JWDL, Ojanen TT (2016). Identity management and sense of belonging to gay community among young rural Thai same-sex attracted men: implications for HIV prevention and treatment. Cult Health Sex.

[89] Walsh CS, Chaiyajit N (2012). Sexperts! Disrupting injustice through HIV prevention and legal rights education with transgenders in Thailand. Digital Cult Educ.

[90] Winter S, Lin AMY (2008). Language and identity in transgender: gender wars, anatomania, and the Thai kathoey. Problematizing identity: everyday struggles in language, culture, and education.

[91] Winter S, Udomsak N. Male, female and transgender: stereotypes and self in Thailand. Int J Transgend. 2002;6(1). https://psycnet.apa.org/record/2002-14060-003.

[92] Yadegarfard M, Ho R, Bahramabadian F (2013). Influences on loneliness, depression, sexual-risk behaviour and suicidal ideation among Thai transgender youth. Cult Health Sex.

[93] Yadegarfard M, Mallika EMB, Ho R (2014). Family rejection, social isolation, and loneliness as predictors of negative health outcomes (depression, suicidal ideation, and sexual risk behavior) among Thai male-to-female transgender adolescents. J LGBT Youth.

[94] APCOM (2012). Policy brief: South East Asia legal environments for men who have sex with men and transgender people.

[95] APCOM (2015). PrEParing Asia: a year after–an overview of country-level progresses on the introduction of PrEP to MSM a year after the APCOM-led Asia-Pacific Regional Consultation on the new HIV prevention tool.

[96] APF & UNDP (2016). Promoting and protecting human rights in relation to sexual orientation, gender identity and sex characteristics: a manual for national human rights institutions.

[97] APTN (2017). From barriers to bridges: increasing access to HIV and other health services for trans people in Asia.

[98] Beyrer C, Wirtz AL, Walker D, Johns B, Sifakis F, Baral SD (2011). The global HIV epidemics among men who have sex with men.

[99] Cameron L (2006). Sexual health and rights: sex workers, transgender people and men who have sex with men—Thailand.

[100] Center for Health Policy Studies, Mahidol University (2016). Review of implementation of comprehensive sexuality education, Thailand.

[101] Godwin J (2010). Legal environments, human rights and HIV responses among men who have sex with men and transgender people in Asia and the Pacific: an agenda for action.

[102] ILO (2005). Equality & discrimination: combating the worst forms of child labour in shrimp and seafood processing areas of Thailand THA/10/50/USA.

[103] Kaleidoscope Human Rights Foundation (2015). Shadow report to the UN Committee on economic, social and cultural rights regarding Thailand's protection of the rights of LGBTI persons.

[104] Kaleidoscope Human Rights Foundation (2016). Report on Thailand regarding the human rights of LGBTI persons: 25th session of the universal periodic review April – may 2016.

[105] Mahidol University (2014). Bullying targeting secondary school students who are or are perceived to be transgender or same-sex attracted: types, prevalence, impact, motivation and preventive measures in 5 provinces of Thailand.

[106] Newman PA, Prabhu SM, Tepjan S, Boborakhimov S, UNICEF East Asia and Pacific Regional Office & United Nations Asia Pacific Interagency Task Team on Young Key Populations. Looking out for adolescents and youth from key populations—Formative assessment of needs for adolescents and youth at risk of HIV: case studies from Indonesia, the Philippines, Thailand and Viet Nam. 2019. https://www.unicef.org/eap/reports/looking-out-adolescents-and-youth-key-populations. Accessed 30 Dec 2020.

[107] NHRC. Evaluative report on the human rights situation in the field of sexual diversity in the B.E. 2547–2549 years (draft): National Human Rights Commission; 2007.

[108] OHCHR (2011). The universal periodic review of the Kingdom of Thailand: issues related to sexual orientation and gender identity.

[109] OHCHR (2018). Revealing the rainbow: the human rights situation of Southeast Asia’s LGBTIQ communities and their defenders.

[110] Prachatai. Is being LGBTIQ psychological problem, or is Thai school lesson being abnormal? Prachatai.com; 2016.

[111] Samakkeekarom R, Taesombat J. Couple life and family building among Thai LGBT people: types, acceptance and needs. Qual Life Law J. 2013;9(2):115–31.

[112] Suksom A (2020). Operational manual for governmental agencies and officials for gender equality protection according to the Gender Equality Act. B.E. 2558 [2015].

[113] Taengkliang C, Kaewwaen A, Chaiyajit N, Yodmuang W (2015). Stakeholder submission to the universal periodic review (UPR) regarding the protection of the rights of LGBTI persons in Thailand.

[114] Taylor M, Robinson J, Vanaspong C (2017). Rapid assessment report trafficking in persons in Thailand.

[115] The World Bank (2016). Getting back on track: reviving growth and securing prosperity for all—Thailand systematic country diagnostic.

[116] Tinnam C, Omphornuwat K, Duaidee R. Strategic plan for LGBTIQN+ well-being— B.E. 2564–2566: Thai Health Promotion Foundation; 2019.

[117] Togetherness for Equality. Thailand: discrimination and violence against women and LBTI persons–shadow report to the Committee on the Elimination of Discrimination Against Women (CEDAW) for consideration at the 67th session, 2017. 2017. https://tbinternet.ohchr.org/Treaties/CEDAW/Shared%20Documents/THA/INT_CEDAW_NGO_THA_27766_E.pdf. Accessed 7 Jan 2021.

[118] U.S. Department of State (2011). Country reports on human rights practices for 2011: Thailand.

[119] U.S. Department of State (2012). Country reports on human rights practices for 2012: Thailand.

[120] U.S. Department of State (2014). Country reports on human rights practices for 2014: Thailand..

[121] UNDP & ILO (2018). LGBTI people and employment: discrimination based on sexual orientation, gender identity and expression, and sex characteristics in China, the Philippines and Thailand.

[122] UNDP & Ministry of Social Development and Human Security (2018). Legal gender recognition in Thailand: a review on laws and policies.

[123] UNDP & USAID (2014). Being LGBT in Asia: Thailand country report.

[124] UNDP (2020). Stories of Stigma: exploring stigma and discrimination against Thai transgender people while accessing health care and in other settings..

[125] UNESCO (2018). School-related violence and bullying on the basis of Sexual Orientation and Gender Identity or Expression (SOGIE).

[126] USAID & UNDP (2011). Men who have sex with men and transgender populations multi-city initiative: city scans and action planning meeting report.

[127] van Wijngaarden JWDL (2016). Changing gears: a guide to effective HIV service programming for gay men and other men who have sex with men in Asia.

[128] Vanaspong C, Kawesri P (2020). Assessment report: implementation of the Gender Equality Act B.E. 2558 (2015).

[129] Winter S, Davis-McCabe C, Russell CB, Wilde D, Chu TH, Suparak AP (2018). Denied work—an audit of employment discrimination on the basis of gender identity in South-East Asia.

[130] Meyer IH (2003). Prejudice, social stress, and mental health in lesbian, gay, and bisexual populations: conceptual issues and research evidence. Psychol Bull.

[131] Fongkaew K, de Lind van Wijngaarden JW, Tepjan S, Chonwanarat N, Akkakanjanasupar P, Newman PA. ‘No test, no disease’: multilevel barriers to HIV testing among young men who have sex with men and young transgender women in three semiurban areas in Thailand. Cult Health Sex. 2021:1–16. [epub ahead of print]. 10.1080/13691058.2021.1938237. 10.1080/13691058.2021.1938237 34254893

[132] Coulter RW, Kenst KS, Bowen DJ (2014). Research funded by the National Institutes of Health on the health of lesbian, gay, bisexual, and transgender populations. Am J Public Health.

[133] Elze DE, Meezan W, Martin JI (2009). Strategies for recruiting and protecting gay, lesbian, bisexual, and transgender youths in the research process. Handbook of research with lesbian, gay, bisexual, and transgender populations.

[134] LaVaccare S, Diamant AL, Friedman J, Singh KT, Baker JA, Rodriguez TA, Cohen SR, Dary FY, Pregler J (2018). Healthcare experiences of underrepresented lesbian and bisexual women: a focus group qualitative study. Health Equity.

[135] Logie CH, James L, Tharao W, Loutfy MR (2012). “We don't exist”: a qualitative study of marginalization experienced by HIV-positive lesbian, bisexual, queer and transgender women in Toronto, Canada. J Int AIDS Soc.

[136] Renn KA (2010). LGBT and queer research in higher education: the state and status of the field. Educ Researcher.

[137] Stall R, Matthews DD, Friedman MR, Kinsky S, Egan JE, Coulter RW (2016). The continuing development of health disparities research on lesbian, gay, bisexual, and transgender individuals. Am J Public Health.

[138] Jackson PA (2000). An explosion of Thai identities: global queering and re-imagining queer theory. Cult Health Sex..

[139] De Schutter O. International human rights law: Cambridge University Press; 2019. 10.1017/9781108564588.

[140] McHugh ML (2012). Interrater reliability: the kappa statistic. Biochem Med (Zagreb).

