# Supplementary material for: LGBT+ inclusion and human rights in Thailand: a scoping review of the literature
Source: BMC Public Health. 2021 Oct 9;21:1816. doi: 10.1186/s12889-021-11798-2 (PMC8501542; doi:10.1186/s12889-021-11798-2)
Supplement: Supplementary file 1 — Additional file 1. [file 12889_2021_11798_MOESM1_ESM.docx]

**Additional File 1**. List of Grey Literature Websites Searched

1. Anjaree Group - anjareefoundation.wordpress.com
2. Apcom – apcom.org
3. Asia Pacific Transgender Network – weareaptn.org
4. Bangkok Rainbow Organization - bangkokrainbow.org
5. Equal Asia Foundation - equalasiafoundation.org
6. Human Rights Watch – hrw.org
7. International Labour Organization – ilo.org
8. International Lesbian, Gay, Bisexual, Trans and Intersex Association – ilga.org
9. Ministry of Social Development and Human Security "m-society.go.th" & "dcy.go.th" & "http://dep.go.th" & "web.codi.or.th"
10. MPLUS FOUNDATION - mplusthailand.com
11. National Human Rights Commission Thailand "nhrc.or.th"
12. Office of the High Commissioner on Human Rights – ohchr.org
13. Rainbow Sky Association of Thailand - rsat.info
14. Teeranat Kanjanauksorn Foundation - teeranat.com
15. Thai Ministry of Justice - Rights and Liberties Protection Department - "moj.go.th"
16. Thai Transgender Alliance - namati.org
17. United Nations – un.org
18. UNDP Thailand –
19. USAID – usaid.gov
20. US State Department – state.gov
21. Women’s Health Advocacy Foundation - whaf.or.th
22. World Bank – worldbank.org
