# Supplementary material for: LGBT+ inclusion and human rights in Thailand: a scoping review of the literature
Source: BMC Public Health. 2021 Oct 9;21:1816. doi: 10.1186/s12889-021-11798-2 (PMC8501542; doi:10.1186/s12889-021-11798-2)
Supplement: Supplementary file 2 — Additional file 2. [file 12889_2021_11798_MOESM2_ESM.docx]

**Additional File 2.** A sample search string for OVID databases

| Thai*.ab,ti. and (((bicurious or bisexual or kathoeys or (sao praphet song or phuying praphet song or toot or tootsie or phet tee sam or tom or dee)).ab,ti. or bisexuality/ or bisexuality.ab,ti. or bisexuals.ab,ti. or cross sex.ab,ti. or crossgender.ab,ti. or F2M.ab,ti. or female-to-male.ab,ti. or gay.ab,ti. or gays.ab,ti. or gender change.ab,ti. or gender dysphoria.ab,ti. or gender identity.ab,ti. or gender queer.ab,ti. or gender reassign.ab,ti. or gender transform.ab,ti. or gender transition.ab,ti. or genderqueer.ab,ti. or GLB.ab,ti. or GLBQ.ab,ti. or GLBs.ab,ti. or GLBT.ab,ti. or GLBTQ.ab,ti. or heteroflexible.ab,ti. or homosexual.ab,ti. or homosexualities.ab,ti. or homosexuality/ or homosexuality.ab,ti. or homosexuals.ab,ti. or intersex.ab,ti. or lesbian.ab,ti. or lesbianism.ab,ti. or lesbians.ab,ti. or lesbigay.ab,ti. or LGB.ab,ti. or LGBQ.ab,ti. or LGBS.ab,ti. or LGBT.ab,ti. or M2F.ab,ti. or male-to-female.ab,ti. or men who have sex with men.ab,ti. or msm.ab,ti. or queer.ab,ti. or same gender loving.ab,ti. or same sex attracted.ab,ti. or same sex couple.ab,ti. or same sex couples.ab,ti. or same sex relations.ab,ti. or sex change.ab,ti. or sex reassign.ab,ti. or sex reversal.ab,ti. or sex transform.ab,ti. or sex transition.ab,ti. or (sexual and gender minorities).ab,ti. or (sexual and gender minority).ab,ti. or sexual identity.ab,ti. or sexual minorities.ab,ti. or sexual minority.ab,ti. or sexual orientation.ab,ti. or sexual preference.ab,ti. or trans female.ab,ti. or trans male.ab,ti. or trans man.ab,ti. or trans men.ab,ti. or trans people.ab,ti. or trans person.ab,ti. or trans woman.ab,ti. or trans-sexuality.ab,ti. or transexual.ab,ti. or transgender.ab,ti. or transgendered.ab,ti. or transgenders.ab,ti. or transsexual.ab,ti. or transsexualism/ or transsexualism.ab,ti. or transsexuality.ab,ti. or transsexuals.ab,ti. or transvestite.ab,ti. or women loving women.ab,ti. or women who have sex with women.ab,ti. or WSW.ab,ti.) not (laparoscopic gastric bypass.ab,ti. or markov state model.mp. or multiple source method.ab,ti.)) |
| --- |
